# Effect of Vagal Nerve Blockade on Moderate Obesity with an Obesity-Related Comorbid Condition: the ReCharge Study

**DOI:** 10.1007/s11695-016-2143-y

**Published:** 2016-04-05

**Authors:** John M. Morton, Sajani N. Shah, Bruce M. Wolfe, Caroline M. Apovian, Christopher J. Miller, Katherine S. Tweden, Charles J. Billington, Scott A. Shikora

**Affiliations:** Stanford School of Medicine, Stanford, CA USA; Tufts Medical Center, Boston, MA USA; Oregon Health & Science University, Portland, OR USA; Boston University, Boston, MA USA; 3D Communications, LLC, Raleigh, NC USA; EnteroMedics Inc, St Paul, MN USA; University of Minnesota, Minneapolis, MN USA; Brigham and Women’s Hospital, Boston, MA USA

**Keywords:** Vagal nerve blocking, Obesity, Moderate obesity, Active implantable medical device, Bariatric surgery, Laparoscopic surgery, Randomized controlled trial

## Abstract

**Background:**

Vagal nerve blockade (vBloc) therapy was shown to be a safe and effective treatment for moderate to severe obesity. This report summarizes the safety and efficacy of vBloc therapy in the prespecified subgroup of patients with moderate obesity.

**Methods:**

The ReCharge Trial is a double-blind, randomized controlled clinical trial of participants with body mass index (BMI) of 40–45 or 35–40 kg/m^2^ with at least one obesity-related comorbid condition. Participants were randomized 2:1 to implantation with either a vBloc or sham device with weight management counseling. Eighty-four subjects had moderate obesity (BMI 35–40 kg/m^2^) at randomization.

**Results:**

Fifty-three participants were randomized to vBloc and 31 to sham. Qualifying obesity-related comorbidities included dyslipidemia (73 %), hypertension (58 %), sleep apnea (33 %), and type 2 diabetes (8 %). The vBloc group achieved a percentage excess weight loss (%EWL) of 33 % (11 % total weight loss (%TWL)) compared to 19 % EWL (6 % TWL) with sham at 12 months (treatment difference 14 percentage points, 95 % CI, 7–22; *p* < 0.0001). Common adverse events of vBloc through 12 months were heartburn/dyspepsia and implant site pain; the majority of events were reported as mild or moderate.

**Conclusions:**

vBloc therapy resulted in significantly greater weight loss than the sham control among participants with moderate obesity and comorbidities with a well-tolerated safety profile.

## Introduction

The vagal nerve blockade (vBloc) device was developed to provide an alternative to standard bariatric surgery for patients with BMI of 35 to 45 kg/m^2^. Placement of the device is by standard laparoscopic surgical techniques without anatomic alterations of the gastrointestinal tract. Development of vagal blocking was based on prior observations that vagotomy could improve obesity [1], including hypothalamic damage obesity [2], and is thought to help patients control feelings of hunger. Randomized clinical trials have demonstrated clinically meaningful weight loss with vBloc therapy [3–5] and improvement in obesity-related comorbid conditions such as type 2 diabetes mellitus (DM2) [6]. Vagal blockade using the vBloc Maestro System is the first new obesity treatment device to receive FDA approval in 14 years.

Weight loss and safety results from the ReCharge randomized controlled trial, which evaluated vBloc therapy using a rechargeable device, have been reported [4]. Using a mixed-effects analysis of the intent-to-treat population, an estimated mean percentage excess weight loss (%EWL) of 26 % (10 % TWL) for vBloc and 17 % for sham (6 % TWL) (*p* < 0.001) at 12 months was demonstrated.

Patients with moderate obesity, BMI 35–40 kg/m^2^, particularly those with obesity-related comorbid conditions, is a cohort of interest since health care assessment groups have noted lack of clinical evidence in this population [7]. The ReCharge Trial enrolled an appreciable number of participants with moderate obesity, and this report summarizes safety and efficacy data from the trial through 12 months in this patient group to confirm that the moderate BMI group had similar efficacy and safety results to the overall cohort and address the lack of clinical evidence in this population.

## Methods

### Participants

The ReCharge Trial study design and methods have been described previously [4]. The majority of participants were enrolled at eight sites in the USA, with two additional sites in Australia. Participants were eligible for enrollment if their BMI was 40–45 or 35–40 kg/m^2^ with one or more of the following obesity-related comorbidities: type 2 diabetes mellitus, hypertension, dyslipidemia, sleep apnea syndrome, or obesity-induced cardiomyopathy. This report includes only those participants with BMI 35–40 kg/m^2^.

### Study Design

ReCharge is a randomized, double-blinded, sham-controlled trial comparing vBloc to a custom-designed sham device. Randomization occurred at implant in a 2:1 ratio to vBloc and sham in permuted block sizes of 3 or 6 stratified by clinical site and type 2 diabetes mellitus status.

Participants and all follow-up personnel, including the sponsor, were blinded until 12-month visits were completed for all trial participants. The interaction of the surgeon with study participants was limited after randomization until the study was unblinded due to the difference in implantation techniques for the two study arms. The study was not unblinded until all participants finished their 12-month visit and data were verified.

The safety of the study was monitored by an independent data and safety monitoring board, and all serious adverse events (SAEs) were independently adjudicated for relatedness by an independent clinical events committee (CEC). The ethical standards for subject treatment set forth in the Helsinki Declaration of 1975 were followed. Written informed consent was obtained from all study participants. The study received institutional review board (IRB) or ethics committee (EC) approval from Bellberry Limited EC, Scottsdale Clinical Research Institute Scottsdale Healthcare, Tufts Medical Center IRB, Oregon Health & Science University IRB, Mayo Clinic Rochester IRB, Stanford University Medical Center IRB, University of Minnesota IRB, Scripps IRB, and Western IRB. The study was registered on clinicaltrials.gov with the identifier NCT01327976.

### Intervention

The Maestro Rechargeable System consists of two implanted components: (1) leads that are placed around the anterior and posterior vagal trunks near the esophagogastric junction using standard laparoscopic surgery and (2) a rechargeable neuroregulator placed subcutaneously on the thoracic wall [8]. The neuroregulator requires in-home transcutaneous charging approximately two times weekly using a transmit coil placed over the neuroregulator.

The sham arm neuroregulator was custom-designed by the sponsor to dissipate electrical charge at a rate identical to the active device. In the sham device, charge was dissipated into resistors within the sham neuroregulator rather than delivering charge to the vagus. No electrodes were implanted and the abdominal cavity was not entered. Skin incisions, placed where trocars would typically be placed, were made to simulate the laparoscopic procedure and support blinding the participant and the follow-up personnel.

The neuroregulator was programmed in both vBloc and sham groups to deliver at least 12 h of therapy per day. Of note, frequent charging of the devices was required in both groups despite no therapy delivery in the sham group. This was a feature designed to protect study blinding. The protocol prespecified weekly therapy current amplitude level increases over the first month to 6 mA. If needed, the amplitude could be adjusted on an alternate schedule by the follow-up team if the participant experienced any discomfort related to the therapy. All participants were asked to check their battery level daily and to recharge their battery when necessary.

Follow-up visits occurred weekly in the first month, bi-weekly through month 3, and monthly thereafter. Monthly visits were conducted within a 2-week window. All patients participated in a weight management program that typically consisted of a 15-min educational discussion at each visit on healthy food choices, exercise, and behavioral modification by a trained dietitian or a research assistant.

### Study Objectives

The primary 12-month efficacy and safety endpoints of the entire study population have been previously reported [4], and overall study outcomes will continue to be assessed for 5 years. The prespecified coprimary efficacy endpoints were as follows: (1) to compare the mean %EWL of the vBloc and sham groups (in the entire sample, at a superiority margin of 10 %) and (2) to assess whether the percentage of participants in the vBloc group with at least 20 % EWL exceeded 55 % and whether the percentage with at least 25 % EWL exceeded 45 %. The primary safety endpoint was to determine whether the rate of serious adverse events (SAEs) related to the device, implant/revision procedure, or therapy in the vBloc group was less than 15 %.

This report focuses on efficacy and safety, through 12 months, in the moderately obese subgroup with BMI of 35 to 40 kg/m^2^. We also present the mean comparison using percentage total weight loss (%TWL) and various commonly cited %TWL thresholds.

### Statistical Analysis

Mixed-effects regression models were used to conduct analyses on the weight loss data in this moderate BMI cohort using the intention-to-treat (ITT) population [9]. Models were fit with an unstructured covariance matrix and treating time (study visits) as a categorical variable with time-specific contrasts. Data are assumed to be missing at random under these models. Weight loss results are also reported as a complete case analysis.

Weight loss results are not reported using the last observation carried forward (LOCF) method, the primary imputation method for the 12-month results for the entire ITT population [4], due to the limitations in statistical properties of LOCF imputation [9]. All statistical analyses were performed using SAS version 9.3.

## Results

### Participant Baseline Characteristics and Disposition

Of the 162 randomized vBloc participants and 77 sham participants, 53 vBloc and 31 sham participants were moderately obese with comorbidities at baseline. The treatment groups were well balanced on baseline demographics and medical characteristics in the moderately obese subgroup (Table 1). Approximately 80 % of the participants were female, and the mean age was 52, and the mean BMI was 38 kg/m^2^. The most common obesity-related comorbidities were dyslipidemia, hypertension, and obstructive sleep apnea.

**Table 1:** Baseline characteristics by treatment group

Characteristic	vBloc (*n* = 53)	Sham control (*n* = 31)
Demographics		
Women, no. (%)	42 (79)	25 (81)
Age, mean (SD) years	53 (8)	51 (7)
Ethnicity, no. (%)		
Hispanic/Latino	1 (2)	2 (7)
Race, no. (%)		
Caucasian	46 (87)	29 (94)
African-American	4 (8)	1 (3)
Native American	2 (4)	0 (0)
Asian	1 (2)	1 (3)
General medical		
Body size measures at implant, mean (SD)		
Height, cm	170 (10)	170 (10)
Implant weight, kg	104 (12)	107 (11)
BMI, kg/m^2^	38 (2)	38 (1)
Excess weight^a^, kg	35 (6)	36 (5)
Waist circumference, cm	115 (11)	117 (9)
Type 2 diabetes mellitus, no. (%)	4 (8)	3 (10)
Hypertension, no. (%)	31 (59)	18 (58)
Dyslipidemia, no. (%)	37 (70)	24 (77)
Obstructive sleep apnea, no. (%)	16 (30)	12 (39)

^a^Excess weight was calculated as the difference between the weight at the time of implantation and the ideal body weight corresponding to a BMI of 25 kg/m^2^. There were no significant differences in any baseline characteristics between the two treatment groups

**Fig. 1:**
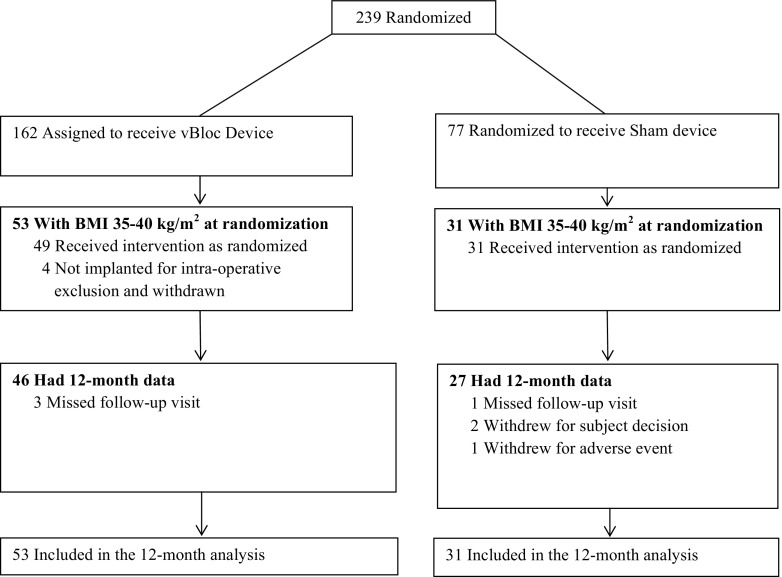
CONSORT diagram of the moderately obese subgroup in the ReCharge Trial

The CONSORT diagram for moderately obese participants in the ReCharge Trial is shown in Fig. 1. Four participants in the vBloc group and three in the sham group had withdrawn from the study by the 12-month visit. The four withdrawals in the vBloc group were due to intra-operative exclusions (discovery of prior upper GI surgery, >5 cm hiatal hernia, cirrhotic liver, and delayed gastric emptying), so the device was not implanted. The three withdrawals in the sham group occurred after implant and were due to breast cancer in one case and subject decision in two cases. One participant in the vBloc group required surgical revision to correct tilt of the neuroregulator within the subcutaneous pocket; the revision was uncomplicated with the patient released on the same day as the procedure. The 12-month visit completion rates were 87 % in both the vBloc and sham groups.

### Weight Loss

**Fig. 2:**
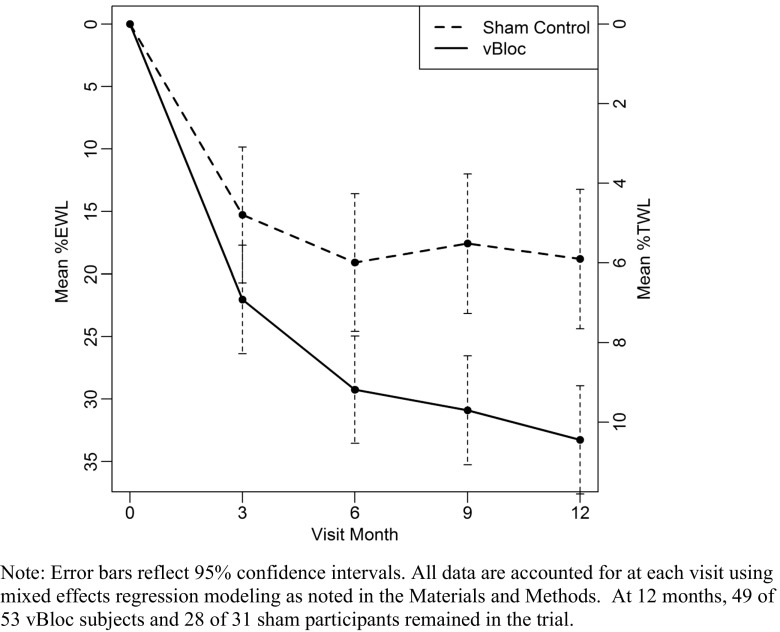
Mean estimated %EWL and %TWL through 12 months

Weight loss expressed as both %EWL and %TWL through 12 months is shown in Fig. 2. At 12 months, the estimated mean %EWL was 33 % (95 % CI, 29–38) in the vBloc group and 19 % (95 % CI, 13–24) in the sham group in the ITT population, with a corresponding treatment difference between groups of 14 percentage points (95 % CI, 7–22, *p* < 0.0001). Estimated mean %TWL was 11 % (95 % CI, 10–12) in the vBloc group and 6 % (95 % CI, 4–8) in the sham group, with a treatment difference of 5 percentage points (95 % CI, 2–7, *p* < 0.0001).

Among the patients completing the 12-month visit (complete case), the mean %EWL was 34 % in the vBloc group and 20 % in the sham group (treatment difference 14 percentage points; 95 % CI, 4–24, *p* = 0.009) and mean %TWL was 11 % in vBloc group and 7 % in the sham group (treatment difference 5 percentage points, 95 % CI, 1–8, *p* = 0.010). The percentage of patients who achieved categorical weight loss thresholds from 20 % EWL to 50 % EWL and from 7.5 % TWL to 15 % TWL is shown in Table 2. The vBloc group had a higher percentage of patients achieve each level of weight loss, and the relative difference between the treatment groups increased as the threshold of weight loss became more difficult to attain. For example, 50 % of vBloc participants achieved at least 30 % EWL compared to 26 % in the sham group; however, 24 % of vBloc participants achieved the higher threshold of at least 50 % EWL compared to 0 % of participants in the sham group.

**Table 2:** Weight loss responder rates in moderately obese subgroup

Weight loss achieved	No. (%)		*p* value
	vBloc *N* = 46	Sham *N* = 27	
% EWL			
≥20 %	33 (72 %)	15 (56 %)	0.16
≥25 %	27 (59 %)	11 (41 %)	0.14
≥30 %	23 (50 %)	7 (26 %)	0.044
≥40 %	18 (39 %)	3 (11 %)	0.011
≥50 %	11 (24 %)	0 (0 %)	0.006
%TWL			
≥7.5 %	31 (67 %)	14 (52 %)	0.187
≥10 %	22 (48 %)	7 (26 %)	0.065
≥15 %	13 (28 %)	2 (7 %)	0.033

Responder rates are presented without imputation

### Safety

The adverse event (AE) profile related to device, procedure, or therapy (Table 3) in this subset of the vBloc group was similar to the entire population at 12 months [4]. Heartburn and dyspepsia, neuroregulator site pain, other pain, abdominal pain, incision pain, nausea, eructation/belching, and dysphagia are the most frequently reported related AEs. More pain at the neuroregulator site was reported in the sham than vBloc group in this subpopulation, though considering the size of the active vBloc and sham devices were identical, this is likely a chance finding due to small sample size. All nonserious AEs in this cohort were reported as mild or moderate in severity, and 84 % of events had resolved at 12 months. Most related events were temporary side effects of the procedure or therapy which resolved with no intervention or with a change in therapy algorithm.

**Table 3:** Adverse events related to device, procedure, or therapy through 12 months

Adverse event	vBloc *N* = 53			Sham *N* = 31		
	No. (%) of patients	No. of events	% events Mild/moderate Severity	No. (%) of patients	No. of events	% events Mild/moderate Severity
Pain, neuroregulator site	15 (28)	17	100	17 (55)	17	100
Other	12 (23)	16	100	3 (10)	3	100
Heartburn/dyspepsia	13 (25)	13	100	1 (3)	1	100
Pain, other	7 (13)	9	100	0 (0)	0	
Pain, abdominal	5 (9)	7	100	0 (0)	0	
Incision pain	5 (9)	6	100	4 (13)	4	100
Nausea	3 (6)	6	100	0 (0)	0	
Eructation/belching	5 (9)	5	100	0 (0)	0	
Dysphagia	4 (8)	4	100	0 (0)	0	
Chest pain	3 (6)	3	100	0 (0)	0	
Constipation	3 (6)	3	100	3 (10)	3	100
Cramps, abdominal	3 (6)	3	100	0 (0)	0	
Dizziness	3 (6)	3	100	0 (0)	0	

Only adverse events attributed by the investigator to the device, procedure, or therapy that occurred in at least 5 % of vBloc group participants are displayed

No primary safety endpoint-related SAEs occurred in the moderately obese vBloc participants through 12 months. Three SAEs (5.7 %) were adjudicated as related to general intra-abdominal surgery in the vBloc arm. Two events were mild or moderate events of nausea due to anesthesia and one event was discovery of a cirrhotic liver at implant, so the participant was not implanted.

## Discussion

In the subset of participants with a BMI from 35 to 40 kg/m^2^ and at least one obesity-related comorbid condition in the ReCharge Trial, weight loss with vBloc therapy was shown to be superior to a rigorous sham control. The vBloc group demonstrated an average weight loss of 33 % EWL or 11 % TWL, with half of participants achieving at least 30 % EWL and one in four patients achieving at least 50 % EWL. In addition, vBloc therapy had a low rate of associated risks. No primary safety endpoint-related events occurred, and all nonserious AEs were mild to moderate in severity. Most AEs spontaneously resolved or were addressed with modification of the therapy algorithm.

With the combination of clinically meaningful weight loss and favorable safety profile in the moderately obese population, vBloc is a reasonable next step after diet and exercise have failed, or for patients not willing to undergo, or not appropriate candidates for, more aggressive surgical interventions [10–12]. There is currently a treatment gap between those patients who can be successful with diet and exercise and those willing to undergo surgery, so additional efficacious and safe treatment options for obesity and comorbidities are needed. While longer-term efficacy data need to be further studied, the 1-year weight loss reported provides support that vagal blockade may be considered an effective alternative to conventional weight loss surgery particularly in this moderate obesity patient group.

Weight loss in the sham group was greater than expected, which has been discussed elsewhere [4]. Briefly, it was hypothesized that the sham response was due to the robust placebo effect of sham surgery along with daily monitoring and recharging of the sham device. In keeping with our dietary intervention studies, the sham effect was not found to persist in the entire cohort of the ReCharge Trial through 18 months as the sham group regained 40 % of the weight lost at 12 months [5].

The analysis in this report has limitations. First, this is an analysis of a subgroup of the entire randomized sample and has limitations inherent to subpopulations. On the other hand, this is offset somewhat by the fact that this subgroup analysis was prespecified, the analysis was conducted using the ITT population, and there were little missing data. Second, analysis of a subgroup diminishes the statistical power to identify significant differences between the treatment groups; however, statistical superiority in mean weight loss was achieved and most weight loss thresholds were met despite this reduced power. Third, results for this subgroup are presented at 12 months, so the durability of weight loss in this subset of participants requires additional follow-up. Results of the entire ReCharge cohort through 18 months have been published [5] and show that the weight loss among vBloc participants were largely sustained, whereas the sham group regained nearly half of the weight they had lost through 12 months despite remaining blinded through most of the period between 12 and 18 months.

## Conclusions

Individuals with moderate obesity have been considered well-suited candidates for vBloc therapy given that the minimally invasive procedure does not require permanent changes to gastrointestinal anatomy, is reversible, and appears to have a more favorable safety profile to traditional weight loss surgery. In this moderately obese population, the ReCharge Trial demonstrated superior weight loss in the vBloc therapy group than the sham group among participants with moderate obesity. Therapy with vBloc is safe and well tolerated. Additional long-term data and continued follow-up of the ReCharge study are needed to further characterize the safety and effectiveness profile of vBloc therapy in subjects with moderate obesity.
